# Eastern Africa Origin of SAT2 Topotype XIV Foot-and-Mouth Disease Virus Outbreaks, Western Asia, 2023

**DOI:** 10.3201/eid3102.240395

**Published:** 2025-02

**Authors:** Antonello Di Nardo, Andrew E. Shaw, Mathilde Gondard, Jemma Wadsworth, Guillaume Girault, Krupali Parekh, Anna Ludi, Valerie Mioulet, Cindy Bernelin-Cottet, Hayley M. Hicks, Noemi Polo, Abdulnaci Bulut, Unal Parlak, Daniel Gizaw, Mustafa Ababneh, Maisa Al Ameer, Layth M.S. Abdulrasool, Fajur S. Al Saloom, Wafa A. Al-Rawahi, Nick J. Knowles, Labib Bakkali-Kassimi, Donald P. King

**Affiliations:** The Pirbright Institute, Pirbright, UK (A. Di Nardo, A.E. Shaw, J. Wadsworth, K. Parekh, A. Ludi, V. Mioulet, H.M. Hicks, N. Polo, N.J. Knowles, D.P. King); ANSES Laboratory for Animal Health, Maisons-Alfort, France (M. Gondard, G. Girault, C. Bernelin-Cottet, L. Bakkali-Kassimi); Foot and Mouth Disease Institute, Ankara, Turkey (A. Bulut, U. Parlak); Animal Health Institute, Sebeta, Ethiopia (D. Gizaw); Jordan University of Science and Technology, Irbid, Jordan (M. Ababneh); Animal Health Laboratory Directorate, Amman, Jordan (M. Al Ameer); Central Veterinary Laboratories, Baghdad, Iraq (L.M.S. Abdulrasool); Ministry of Municipalities Affairs and Agriculture, Hawrat A'ali, Bahrain (F.S. Al Saloom); Sultan Qaboos University, Muscat, Oman (W.A. Al-Rawahi); Central Laboratory of Animal Health, Muscat (W.A. Al-Rawahi)

**Keywords:** foot-and-mouth disease, serotype SAT2, molecular epidemiology, real-time RT-PCR, viruses, eastern Africa, western Asia

## Abstract

We describe detection of SAT2 topotype XIV foot-and-mouth disease viruses in western Asia during 2022–2023. Sequences show the viruses originated in eastern Africa and were introduced into western Asia on >1 occasion. The rapid spread in naive animals highlights risks for onward transmission and potential endemicity in Asia.

Cases of foot-and-mouth disease (FMD) were reported in buffalo in Baghdad, Iraq, during December 2022 (https://wahis.woah.org/#/in-review/4856). Genotyping of FMD virus (FMDV) variable protein (VP) 1 coding sequences generated by the SAP Institute in Turkey identified the causative FMDV as the SAT2/XIV topotype that is present in eastern Africa but is exotic to western Asia (*1*). Increased surveillance monitored the further spread of this topotype among naive livestock that had not been vaccinated or previously infected with this serotype. By January 2023, fresh outbreaks caused by SAT2/XIV were detected in Bahrain, Jordan, and Oman. In March 2023, outbreaks caused by this topotype were also reported in eastern Anatolia in Turkey and later in central Anatolia and the Adana Provinces. We used whole-genome sequences to investigate the likely timing and route of SAT2/XIV incursion and spread in western Asia.

## The Study

We performed whole-genome sequencing of 49 SAT2/XIV FMDV isolates from clinical samples submitted to the World Reference Laboratory for FMD (WRLFMD; Pirbright, UK) or the French Agency for Food, Environmental and Occupational Health and Safety (ANSES; Paris, France). Specifically, sequences consisted of 12 samples from Ethiopia collected during May 2022–January 2023, six samples from Iraq collected during December 2022–February 2023, six samples from Jordan collected during January–February 2023, three samples collected in Bahrain in November 2021, seventeen samples from Turkey collected during March–June 2023 (all submitted to WRLFMD), and 5 samples from Oman collected during January–February 2023 (submitted to ANSES) (Appendix Table 1). Sequences were determined at WRLFMD using Illumina MiSeq technology (https://www.illumina.com) as previously described (*2*) and at ANSES using the Oxford Nanopore MinION platform (https://www.nanoporetech.com) (in-house protocol).

**Figure 1:**
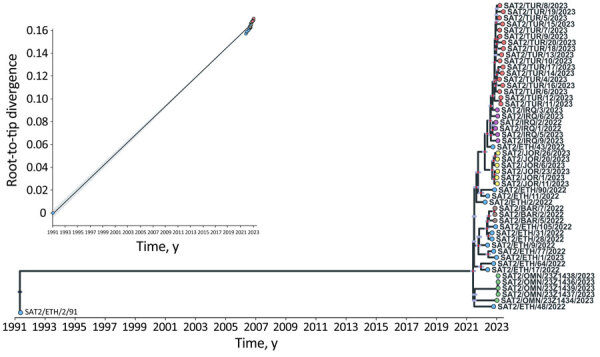
Time-scaled phylogeny reconstructed from whole genomes of the type SAT2 topotype XIV foot-and-mouth disease virus in study of eastern Africa origin of SAT2 topotype XIV foot-and-mouth disease virus outbreaks, western Asia, 2023. Tips are colored according to the country of isolation. Internal nodes colored in red identify those clades resulting with a posterior probability of >0.75. The 95% Bayesian credible interval reporting uncertainty region in the timing of each ancestral node is represented by the light blue horizontal bars. The inset shows the correlation of the SAT2 topotype XIV root-to-tip divergence with the time of sample collection. BAR, Bahrain; ETH, Ethiopia; IRQ, Iraq; JOR, Jordan; OMN, Oman; TUR, Turkey.

**Figure 2:**
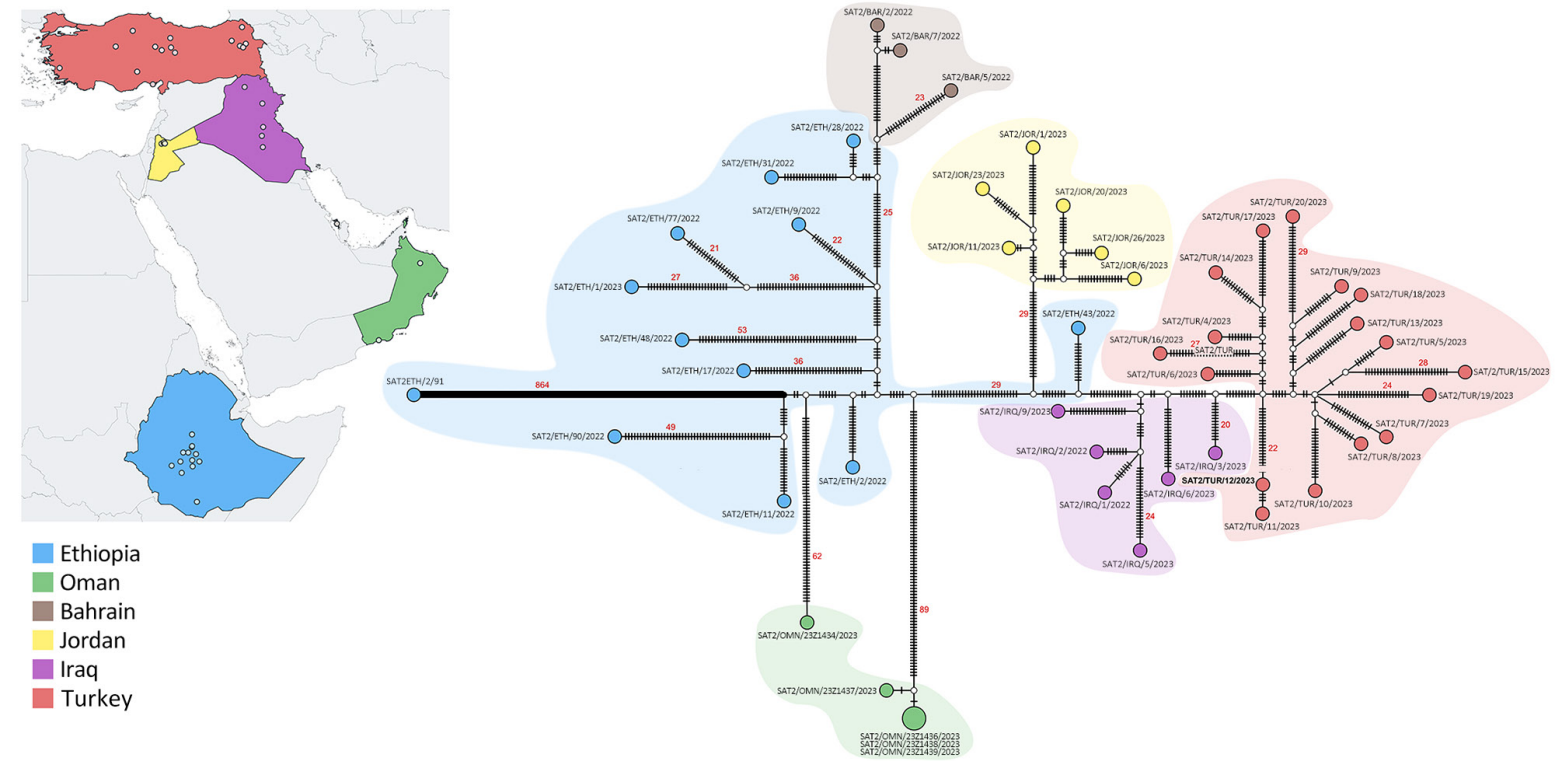
Statistical parsimony network and geographic distribution of type SAT2 topotype XIV foot-and-mouth disease viruses in study of eastern Africa origin of SAT2 topotype XIV foot-and-mouth disease virus outbreaks, western Asia, 2023. Nodes are colored according to the country of isolation; white nodes represent missing unsampled haplotypes. Hatch marks represent single nucleotide substitutions estimated between the connected nodes. Differences in nucleotide substitutions of >20 are reported in red numbers. BAR, Bahrain; ETH, Ethiopia; IRQ, Iraq; JOR, Jordan; OMN, Oman; TUR, Turkey.

Bayesian phylogenetic reconstruction (*3*) (Figure 1) demonstrated that the SAT2/XIV FMDV sequences were assigned into distinct sister clades, providing evidence for ancestors circulating in eastern Africa that moved into western Asia causing outbreaks in Iraq, Jordan, and Turkey (most recent common ancestor [MRCA] dated to March 2022 [95% Bayesian credible interval (BCrI) February–May 2022]); and independent introductions into Bahrain (MRCA dated May 2022, 95% BCrI April–June 2022) and Oman (MRCA dated July 2021, 95% BCrI June–September 2021). Those viruses are distantly related (98.70% +13.01 nt identity) to a virus collected during March 2022 from the Wolayita Zone in southwestern Ethiopia. The MRCA of the SAT2/XIV topotype was estimated to be April 1991 (95% BCrI March–May 1991) with an evolutionary rate of 5.46 × 10^−3^ nt/site/year (95% BCrI 4.686.16 × 10^−3^ nt/site/year). To our knowledge, SAT2/XIV was detected on only 1 other occasion, in 1991 on a dairy cattle farm located southwest of Addis Ababa, Ethiopia. However, infrequent sampling makes pinpointing the precise source of these viruses within eastern Africa difficult. Statistical parsimony network analysis (*4*) produced a similar topological representation of the SAT2/XIV FMDV sequences (Figure 2). However, this analysis highlighted several key additional points: infections in Oman were caused by 2 divergent viruses, providing evidence for 2 independent introductions; cases in Bahrain originated from a single virus ancestor, which evolved from viruses circulating in Ethiopia; viruses from Iraq evolved from an eastern Africa ancestor, which also provided the source for onward transmission to Turkey; and the SAT2/XIV outbreaks in Jordan were likely derived from a single independent virus introduction with a different genetic origin than cases reported in Iraq and Turkey.

We observed a single-point mutation at site 8080 (C>T) on the SAT2/TUR/16/2023 genome, shifting the termination to 21 nt downstream of the stop codon at a TGA leading to an extra 7 amino acids (QSLRCHN) on the end of the 3Dpol. A GenBank search of closely related genomes found that this mutation was also present on a SAT2/XIV genome reported separately from Jordan (GenBank accession no. PP112252).

**Figure 3:**
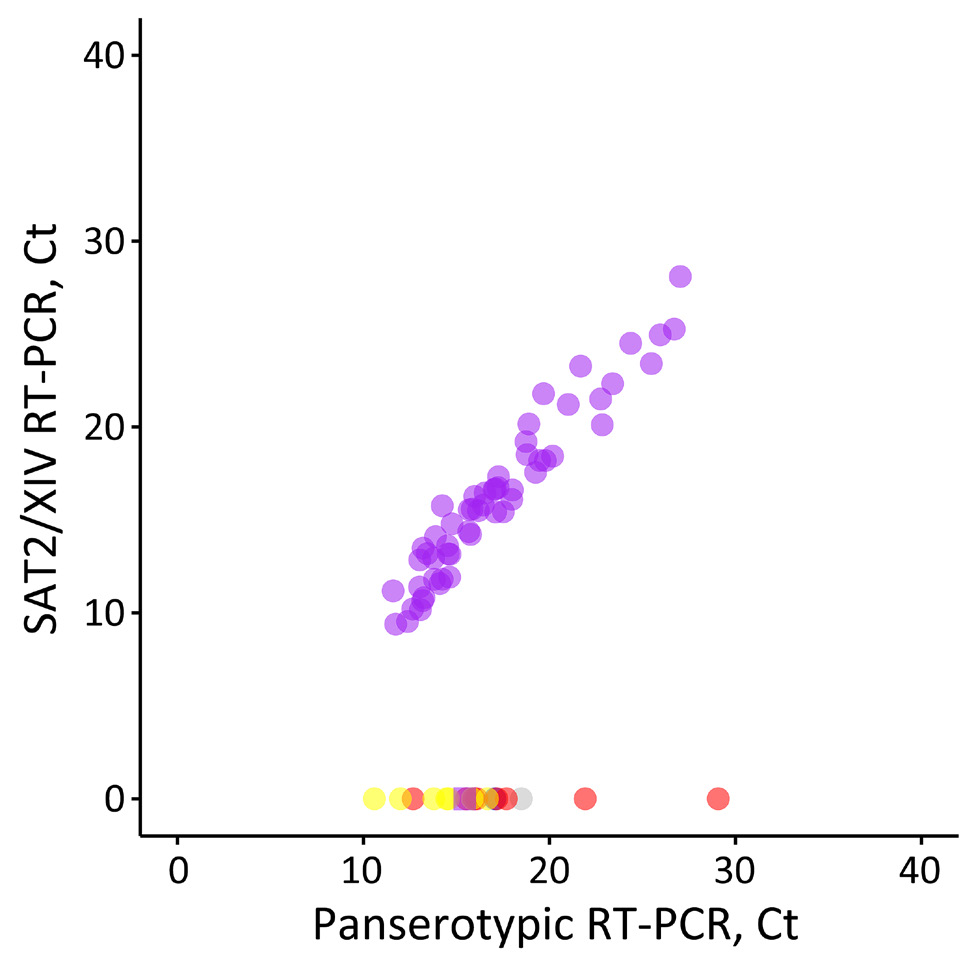
Performance of the SAT2/XIV-specific real-time RT-PCR for foot-and-mouth disease virus–positive samples representing different viral lineages and topotypes that are circulating or threaten countries in western Asia in study of eastern Africa origin of SAT2 topotype XIV foot-and-mouth disease virus outbreaks, western Asia, 2023. Plotted data shows real-time RT-PCR results for the SAT2/XIV specific test compared with results for the pan-serotype (3D) assay performed in parallel (*5*). Samples tested included clinical samples collected from confirmed SAT2/XIV cases (purple, n = 55) and characterized isolates from the O/ME-SA/PanAsia-2, O/ME-SA/Ind-2201 and O/EA-3 lineages (red, n = 8), the A/ASIA/Iran-05 lineage (blue, n = 1), the SAT1/I topotype (yellow, n = 7), the SAT2/VII topotype (purple, n = 8), and serotype Asia 1 (gray, n = 1). Ct, cycle threshold; RT-PCR, reverse transcription PCR.

We designed an FMDV lineage–specific real-time reverse transcription PCR (RT-PCR) assay within the 1D region to detect viruses from the SAT2/XIV topotype (Table). We assessed specificity in silico by using alignments of sequences from other viral lineages circulating in the region (A/ASIA/Iran-05, O/ME-SA/PanAsia-2, Asia1/ASIA/Sindh-08, SAT2/VII) and then through experimental testing using representative samples. The real-time RT-PCR correctly categorized 56 samples as SAT2/XIV with no cross-reactivity observed with isolates of serotypes O, A, Asia1, or the SAT1/I and SAT2/VII topotypes (Figure 3).

**Table:** Primers and probes designed for SAT2/XIV topotype-specific real-time reverse transcription PCR in study of east Africa origin of SAT2 topotype XIV foot-and-mouth disease virus outbreaks, western Asia, 2023*

Oligo name	Nucleotide sequence, 5′ → 3′	Genome location	Use
SAT2_XIV_AS_P	CCTCCACTGCCATCCGCGGTGAYAGG	3663–3688	Probe
SAT_XIV_AS_F	ACCGTGTACAACGGTGAGTG	3629–3648	Forward primer
SAT2_XIV_AS_R	TCAGCGTACTTGGCCRCAAG	3714–3695	Reverse primer

*FMDV genome locations are based on the ETH/2/2002 full genome sequence (GenBank accession no. OQ557397).

We obtained evidence of antigen match of SAT2 vaccine strains against SAT2/XIV field viruses using 2D-VN testing (*6*); all (11/11) isolates were matched to the SAT2 Eritrea 98 vaccine, whereas 8 of the isolates were matched to the SAT2 ZIM 83 vaccine. Although no vaccine-protection data are available for SAT2/XIV viruses, the mean heterologous log_10_ titers (1.65 + 0.12 for the SAT2 Eritrea 98 vaccine and 1.97 + 0.17 for the SAT2 ZIM 83 vaccine) for bovine serum samples exceeded the cutoff of log_10_ 1.5 previously defined in a SAT 2 potency study for a SAT2/VII isolate (*7*) (Appendix Table 2).

## Conclusions

We report the incursion of the FMDV SAT2/XIV topotype into western Asia and used whole-genome sequencing to reconstruct virus movements from eastern Africa. The emergence of exotic serotype SAT2, and the rapidity with which this virus lineage spread among a naive population, poses threats to the region, as well as to countries in Europe protected by the vaccination buffer zone in Turkish Thrace (*8*).

FMDV epidemiology is documented by dynamic cross-exchange and long-distance movements of viruses between the 7 endemic pools (*9*,*10*). Escapes of FMDV lineages endemic in Africa have been recorded for types A, O, SAT1, and SAT2 viruses; documented examples include SAT1 topotype VI in Bahrain (1962), Greece (1962), Iran (1962–1964), Iraq (1962), Israel (1962), Jordan (1962), Lebanon (1962), Syria (1962), and Turkey (1962–1965) (*11*). SAT1 was detected on 2 further occasions: an unknown genotype in Kuwait (1969–1970) and Saudi Arabia (1970) (12) and topotype VI in Yemen (1984) (N.J. Knowles, unpub. data). Serotype O, topotype EA-3, was recorded in Yemen during 1971–2009 and more recently in Israel (2017) and Palestine (2017–2018) (*13*). Serotype A topotype AFRICA lineages have been detected: G-II in Yemen (1985), G-VII in Yemen (1989 and 1998), and G-I in Oman (2018–2021) and Bahrain (2021) (*14*). Serotype SAT2 lineages have been reported on 6 occasions: topotype IV in Yemen (1990) and Bahrain (2012) and topotype VII in Kuwait (2000), Saudi Arabia (2000), Palestine (2012), and Oman (2015) (*15*). Those events highlight epidemiologic connections between eastern Africa and western Asia.

Incursions of exotic FMDVs into new areas, and especially from eastern Africa, have been associated with trade in livestock or products of animal origins (*10*). A recent risk assessment identifies likely pathways of SAT2/XIV diffusion within western Asia, which points to the risk posed by informal livestock trade routes and common grazing (*8*). Islamic festivals, such as Eid al-Adha, increase demand for meat across the region driving differential pricing, which could also potentially affect the epidemiology of FMD (https://cadmus.eui.eu/handle/1814/75333).

Although data are derived from opportunistic sampling, our findings support 2 hypotheses. First, recent introductions of SAT2/XIV into western Asia are defined by multiple independent incursions; furthermore, the outbreaks detected in Oman and Bahrain appear not to be directly linked, suggesting independent introductions of the virus could have occurred during 2022–2023. Second, cases in Iraq were caused by viruses derived from a single ancestor introduced during late 2022, and the cases subsequently detected in Turkey likely originated from Iraq; however, cases reported in Jordan are likely caused by a virus of a different origin. Uncertainties remain surrounding the origin of SAT2/XIV and how this topotype has been maintained during the 10 years before its reappearance in 2022 in eastern Africa, although a wildlife reservoir, likely within Cape buffalo populations, might represent its ecologic niche.

Appendix Additional information about eastern Africa origin of SAT2 topotype XIV foot-and-mouth disease virus outbreaks, western Asia, 2023.
