# Supplementary material for: Eastern Africa Origin of SAT2 Topotype XIV Foot-and-Mouth Disease Virus Outbreaks, Western Asia, 2023
Source: Emerg Infect Dis. 2025 Feb;31(2):368–72. doi: 10.3201/eid3102.240395 (PMC11845145; doi:10.3201/eid3102.240395)
Supplement: Appendix — Additional information about eastern Africa origin of SAT2 topotype XIV foot-and-mouth disease virus outbreaks, western Asia, 2023. [file 24-0395-Techapp-s1.pdf]

Article DOI: https://doi.org/10.3201/eid3102.240395

EID cannot ensure accessibility for supplementary materials supplied by authors. Readers who have difficulty accessing supplementary content should contact the authors for assistance.

East African Origin of SAT2 Topotype XIV Foot-and-Mouth Disease Virus Outbreaks, Western Asia, 2023

Appendix

Appendix Table 1. Details of the 49 FMDV isolates collected from SAT2/XIV outbreaks used for whole-genome sequence analysis

Virus designation	Country	Location	Species	Date collected	GenBank accession no.
ETH/2/91	Ethiopia	Stella Farm, Addis Ababa	Cattle	1991	OQ557396
ETH/2/2022	Ethiopia	Sodo, Wolaita, SNNPR	Cattle	29/03/2022	OQ557397
ETH/9/2022	Ethiopia	Gibe, Hadiya, SNNPR	Cattle	28/05/2022	PQ587563
ETH/11/2022	Ethiopia	Dedo, Jima, Oromia	Cattle	31/05/2022	PQ587564
ETH/17/2022	Ethiopia	Goro, Southwest Shoa, Oromia	Cattle	31/05/2022	PQ587565
ETH/28/2022	Ethiopia	Degam, North Shoa, Oromia	Cattle	14/09/2022	PQ587566
ETH/31/2022	Ethiopia	Degam, North Shoa, Oromia	Cattle	14/09/2022	PQ587567
ETH/43/2022	Ethiopia	Arero, Borana, Oromia	Cattle	05/10/2022	PQ587568
ETH/48/2022	Ethiopia	Gidu kombolcha, East Shoa, Oromia	Cattle	22/10/2022	PQ587569
ETH/64/2022	Ethiopia	Alemtena, East Shoa, Oromia	Cattle	22/10/2022	PQ587570
ETH/77/2022	Ethiopia	Negele Arsi, East Shoa, Oromia	Cattle	24/10/2022	PQ587571
ETH/90/2022	Ethiopia	Seden Sodo, Southwest Shoa, Oromia	Cattle	14/11/2022	PQ587572
ETH/105/2022	Ethiopia	Sebeta, Southwest Shoa, Oromia	Cattle	08/12/2022	PQ587573
ETH/1/2023	Ethiopia	Digaluenatijo, Arsi, Oromia	Cattle	01/01/2023	PQ587574
BAR/2/2022	Bahrain	-	Cattle	23/11/2022	PP432642
BAR/5/2022	Bahrain	-	Cattle	24/11/2022	PP432643
BAR/7/2022	Bahrain	-	Cattle	24/11/2022	PP432644
IRQ/1/2022	Iraq	Al-Fudhailia, Baghdad Governorate	Water buffalo	06/12/2022	PP432645
IRQ/2/2022	Iraq	Al-Fudhailia, Baghdad Governorate	Water buffalo	18/12/2022	PP432646
IRQ/3/2023	Iraq	Humidat, Ninevah Governorate	Cattle	18/01/2023	PP432647
IRQ/5/2023	Iraq	Daqooq, Kirkuk Governorate	Cattle	26/01/2023	PP432648
IRQ/6/2023	Iraq	Al-Mahawil, Babil Governorate	Cattle	29/01/2023	PP432649
IRQ/9/2023	Iraq	Al-Mishkhab, Najaf Governorate	Water buffalo	02/02/2023	PP432650
JOR/1/2023	Jordan	Dulil, Zarqa City, Zarqa Governorate	Cattle	08/01/2023	PP432651
JOR/6/2023	Jordan	Zarqa City, Zarqa Governorate	Cattle	15/01/2023	PP432652
JOR/11/2023	Jordan	Dulil, Zarqa City, Zarqa Governorate	Cattle	18/01/2023	PP432653
JOR/20/2023	Jordan	Halabat, Zarqa City, Zarqa Governorate	Cattle	23/01/2023	PP432654
JOR/23/2023	Jordan	Khaldieh, Mafraq City, Mafraq Governorate	Cattle	25/01/2023	PP432655
JOR/26/2023	Jordan	Halabat, Zarqa City, Zarqa Governorate	Cattle	07/02/2023	PP432656
TUR/4/2023	Turkey	Mehmetakifersoy, Merkez, Agri	Cattle	11/03/2023	PP432657
TUR/5/2023	Turkey	Erdal, Tutak, Agri	Cattle	11/03/2023	PP432658
TUR/6/2023	Turkey	Seyhali, Polatli, Ankara	Cattle	17/03/2023	PP432659
TUR/7/2023	Turkey	Degim, Merkez, Cankiri	Cattle	17/03/2023	PP432660
TUR/8/2023	Turkey	Mesudiye, Germencik, Aydin	Cattle	21/03/2023	PP432661
TUR/9/2023	Turkey	Yagan, Koprukoy, Erzurum	Cattle	24/03/2023	PP432662
TUR/10/2023	Turkey	Yukarikarahalit, Tutak, Agri	Cattle	24/03/2023	PP432663
TUR/11/2023	Turkey	Cakiroren, Karatas, Adana	Cattle	17/04/2023	PP432664
TUR/12/2023	Turkey	Cakiroren, Karatas, Adana	Cattle	17/04/2023	PP432665

Virus designation	Country	Location	Species	Date collected	GenBank accession no.
TUR/13/2023	Turkey	Avsaroren, Kangal, Sivas	Cattle	04/05/2023	PP432666
TUR/14/2023	Turkey	Enidogan, Gemerek, Sivas	Cattle	18/05/2023	PP432667
TUR/15/2023	Turkey	Guneykaya, Yildizeli, Sivas	Cattle	26/05/2023	PP432668
TUR/16/2023	Turkey	Turkmen, Eregli, Konya	Cattle	30/05/2023	PP432669
TUR/17/2023	Turkey	Kurkcu, Sarikaya, Yozgat	Cattle	08/06/2023	PP432670
TUR/18/2023	Turkey	Baharozu, Ulas, Sivas	Cattle	14/06/2023	PP432671
TUR/19/2023	Turkey	Baharozu, Ulas, Sivas	Cattle	14/06/2023	PP432672
TUR/20/2023	Turkey	Buyuksutluce, Merkez, Ardahan	Cattle	26/06/2023	PP432673
OMN/23Z1434/2023*	Oman	Nizwa, Ad Dakhiliyah	Goat	10/01/2023	PP587765
OMN/23Z1436/2023*	Oman	Salalah, Dhofar	Cattle	08/02/2023	PP587766
OMN/23Z1437/2023*	Oman	Salalah, Dhofar	Cattle	08/02/2023	PP587767
OMN/23Z1438/2023*	Oman	Salalah, Dhofar	Cattle	08/02/2023	PP587768
OMN/23Z1439/2023*	Oman	Salalah, Dhofar	Cattle	08/02/2023	PP587769

*Non–World Reference Laboratory for Foot-and-Mouth Disease (Pirbright, UK) reference number.

Appendix Table 2. Vaccine matching data resulting from 2D-VNT testing of representative SAT2/XIV FMDV isolates using bovine reference sera generated from two SAT2 vaccine strains produced by Boehringer Ingelheim. r_1 values express antigenic relationship, as defined by the reactivity of the reference serum against the heterologous (field) strain divided by the reactivity of the serum against the homologous (vaccine) strain. r_1 values of >0.30 (green cells) denote a field virus that is antigenically matched to the FMD vaccine.

	FMD vaccine strain			
	SAT2 Eritrea 98		*SAT2 Zim 83*	
Virus designation	Heterologous titer (log_{10})	r_1	Heterologous titer (log_{10})	r_1
ETH/2/2022	1.62	0.81	2.17	0.54
IRQ/2/2022	1.75	0.73	2.04	0.38
IRQ/5/2023	1.61	0.53	2.33	0.74
IRQ/9/2023	1.51	0.42	1.95	0.31
JOR/11/2023	1.69	0.58	1.74	0.20
JOR/20/2023	1.85	0.83	1.81	0.24
JOR/26/2023	1.86	0.66	1.94	0.32
BAR/2/2022	1.50	0.40	1.85	0.39
BAR/7/2022	1.62	0.53	1.99	0.53
TUR/4/2023	1.68	1.00	1.95	0.31
TUR/17/2023	1.52	0.72	1.86	0.25
